# In Vitro Evaluation of Combined Commercialized Ophthalmic Solutions Against *Acanthamoeba* Strains

**DOI:** 10.3390/pathogens8030109

**Published:** 2019-07-25

**Authors:** María Reyes-Batlle, Glorian Mura-Escorche, Ines Sifaoui, Alejandro Otero-Ruiz, Rosalía Alfaro-Sifuentes, Atteneri López-Arencibia, Pedro Rocha-Cabrera, Olfa Chiboub, Aitor Rizo-Liendo, Jonadab Zamora-Herrera, Carlos J. Bethencourt-Estrella, Rubén L. Rodríguez-Expósito, Desirée San Nicolás-Hernández, José E. Piñero, Jacob Lorenzo-Morales

**Affiliations:** 1 Instituto Universitario de Enfermedades Tropicales y Salud Pública de Canarias, Universidad de La Laguna. Av. Astrofísico Francisco Sánchez S/N, 38203 Tenerife, España; 2 Departamento de Ciencias Agronómicas y Veterinarias, Instituto Tecnológico de Sonora, Cd. Obregón, Sonora 95370, Mexico; 3 Departmento de Oftalmología, Hospital Universitario de Canarias, 38320 La Laguna, Tenerife, Canary Islands, Spain; 4 Laboratoire Matériaux-Molécules et Applications, La Marsa, University of Carthage, Tunisia 1003, Tunisia

**Keywords:** *Acanthamoeba*, keratitis, ophthalmic solution, chemotherapy, amoebicidal activity

## Abstract

*Acanthamoeba* is a free-living amoebae genus which is present worldwide in natural and artificial environments. These amoebae are clinically important as causative agents of diseases in humans and other animals such as a fatal encephalitis or a sight threatening *Acanthamoeba* keratitis (AK). Lately; studies have focused on the search of novel therapeutic options for AK but also to prevent infections. Furthermore; the evaluation of commercialized products seems to be an option for this case since not clinical assays would be required. Thus; we aimed to test the amoebicidal activity of different mixtures of two commercial ophthalmic solutions: Systane^®^ Ultra; which has already shown anti-*Acanthamoeba* properties; and Naviblef^®^ Daily Care. In addition, we tested their cytotoxic effect against murine macrophages. At the individual level; Naviblef^®^ Daily Care showed to be the most active product against *Acanthamoeba* spp. Nevertheless; the combinations of Systane^®^ Ultra and Naviblef^®^ Daily Care; showed an improvement in the activity against trophozoites and cysts of *Acanthamoeba castellanii* Neff. Moreover; the concentration necessary to generate cytotoxic effect against murine macrophages (J774.1) was much higher than the required for the amoebicidal and cysticidal effect achieved in the most effective mixtures.

## 1. Introduction

*Acanthamoeba* is a widely distributed protozoa which has been isolated from many sources such as soil, water, contact lenses, air-conditioning units, clinical samples among others [1,2]. The main human pathologies caused by this genus are *Acanthamoeba* Keratitis (AK) and Granulomatous Amoebic Encephalitis (GAE). At the early stage of infection, AK presents symptoms such as eye redness, epithelial defects, photophobia and intense pain, but if no early and accurate diagnosis and effective treatment is performed, it could end in blindness or even in eye removal [2,3,4,5]. Additionally, severity of the infection depends on the *Acanthamoeba* strain, the cornea integrity and the host immune response [6].

Until now, there is no effective therapy for AK, but the recommended treatment regimen for AK includes a biguanide (0.02% polyhexamethylene biguanide (PHMB) or 0.02% chlorhexidine digluconate) accompanied with diamidine (0.1% propamidine isethionate, also known as Brolene, or 0.1% hexamidine, also known as Desomedine) [7,8]. Lately, voriconazole has been demonstrated to be effective against clinical strains of *Acanthamoeba* [9,10] and has also been successfully used in a clinical case of AK in Spain [11]. One of the main problems on AK recovery is the ability of *Acanthamoeba* to form an extremely resistant cyst phase. Therefore, the need for efficient therapies not only against the trophozoite form but also to this resistant stage is evident.

AK mostly affects contact lenses (CL) wearers and the reported cases are mostly related to the use of soft CL and their incorrect use and maintenance [12,13]. Moreover, the use of CL is also associated to other eye diseases, such as dry eye disease (DED) or Blepharitis. DED has been demonstrated as a predisposing factor to develop AK [14].

Systane^®^ Ultra (Systane^®^; Alcon, Fort Worth, TX, USA; Table 1) is one of the most widely used ocular lubricants in the USA [15]. In a recent study, the amoebicidal activity of this product was reported as well as its potential use for the prevention or even treatment of *Acanthamoeba* keratitis, or to contribute for an optimized solution [16]. Naviblef^®^ Daily Care (Novax^®^ Pharma, Monaco; Table 1) is a commercialized cleaning foam which contains tea tree oil (TTO) (0.02%) and chamomile oil (CO). This foam effectively removes excessive oil, pollen and other debris from the eyelids which increase the risk of irritation, blepharitis due to the presence of Demodex or bacteria as well as dry eye symptoms.

Benzalkonium chloride (BAK) or its derivatives such as POLYQUAD^®^ is one of the most frequently used preservatives in ophthalmic solutions. The cytotoxic effects of BAK has been shown to induce inflammation on the ocular surface cells in numerous *in vitro* and *in vivo* models [17]. Systane^®^ Ultra, which contains POLYQUAD^®^, has shown induction of inflammation markers. On the other hand, Naviblef^®^ Daily Care does not present any described toxic preservatives on its composition. In the present study, the *in vitro* anti-*Acanthamoeba* effect of different combinations of Systane^®^ Ultra and Naviblef^®^ Daily Care (Table 2) were tested as well as their cytotoxic effect against murine macrophages.

## 2. Results

### 2.1. In Vitro Effect of Systane^®^ Ultra and Naviblef^®^ Daily Care Against the Trophozoite Stage of Acanthamoeba Strains

Both Systane^®^ Ultra and Naviblef^®^ Daily Care presented high anti-trophozoite activity against the three *Acanthamoeba* tested strains (Table 1). According to the present results, the lowest IC_50_ values were the obtained after Naviblef^®^ Daily Care treatment in all strains, ranging from 0.68 ± 0.16%, in *A. castellanii* Neff, to 1.13 ± 0.16%, in *A. griffini.* On the other hand, Systane^®^ Ultra IC_50_ values have varied from 4.97 ± 0.71%, in *A. castellanii* Neff, to 7.99 ± 1.25%, in the case of *A. griffini.*

### 2.2. In Vitro Effect of Systane^®^ Ultra and Naviblef^®^ Daily Care Against the Cyst Stage of A. Castellanii Neff Strain

The colorimetric assay based on the alamarBlue^TM^ reagent demonstrated the cysticidal activity of Systane^®^ Ultra and Naviblef^®^ Daily Care, with IC*_50_* values of 34.6 ± 1.38% and 2.1 ± 0.04% respectively (Table 1).

### 2.3. In Vitro Effect of Systane^®^ Ultra and Naviblef^®^ Daily Care Mixtures

**Figure 1:**
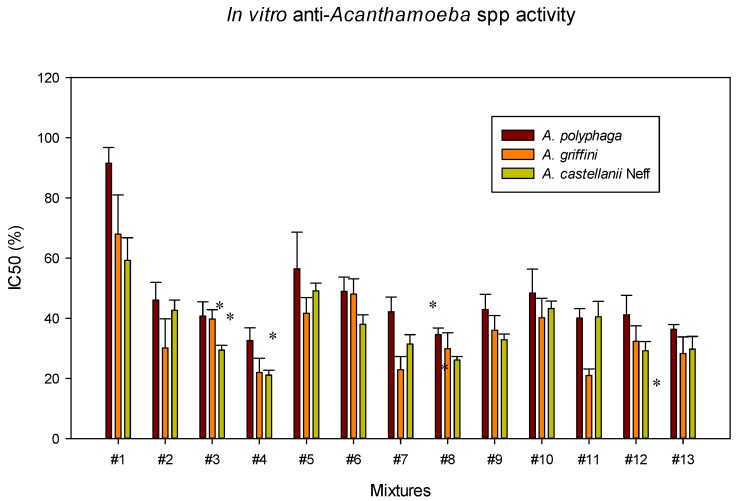
Comparison of the amoebicial effects of mixtures in the three tested strains of *Acanthamoeba*: *A. castellanii* Neff, *A. polyphaga,* and *A. griffini.* Error bars show standard deviations. One Way ANOVA, *p* ˂ 0.050 (*).

**Figure 2:**
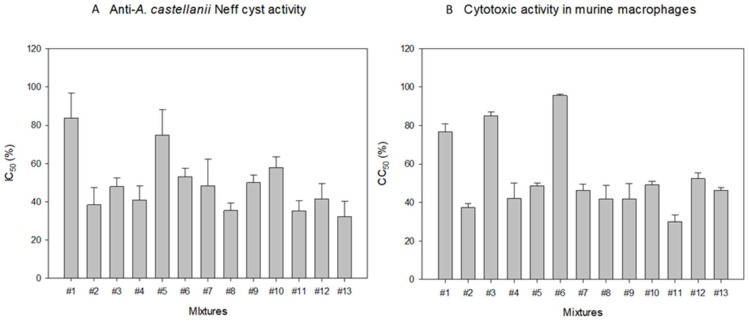
*In vitro* effect of Systane^®^ and Naviblef^®^ Daily Care calculated mixtures against *A. castellanii* cysts (**A**) and murine macrophages J774A.1 (B).

Once the individual *in vitro* activity of each of the ophthalmic solutions was performed, the different combinations obtained with the Design-Expert^®^ software were tested against *Acanthamoeba* spp. (Figure 1), *A. castellanii* Neff cysts and murine macrophages (Figure 2). All mixtures showed activity against *Acanthamoeba* spp., being the highest IC_50_ values the ones for *A. polyphaga* and the lowest ones for *A. castellanii* Neff (Figure 1). In order to compare the *in vitro* activity against *A. castellanii* Neff trophozoites of each ophthalmic solution and their combinations, the percentage of Systane^®^ Ultra and Naviblef^®^ Daily Care was calculated for each mixture for all IC_50_ values (Table 2). Moreover, we noticed a decrease in the quantity of each eye drop solution needed to inhibit 50% of amoebae growth when combinations were used. All IC_50_ values against *A. castellanii* Neff cysts were higher than the IC_50_ against trophozoites except in #2 and #11 (Figure 2), where the Systane^®^ Ultra and Naviblef^®^ Daily Care proportions were 0.43:0.43 (%:%) and 1.52:0.31 (%:%) respectively (Table 2). Both mixtures, #2 and #11 presented the lowest CC_50_ (%) against murine macrophages, 37.39 ± 1.95% and 30.05 ± 3.58% respectively (Figure 2).

### 2.4. Response Surface Analysis

**Figure 3:**
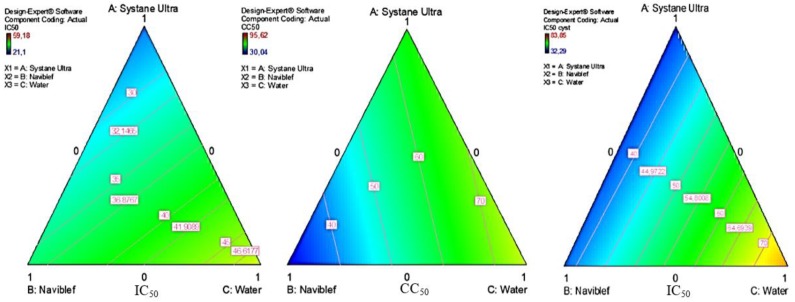
Response surface analysis diagrams. (**A**): IC_50_ in *A. castellanii* Neff trophozoites; (**B**): CC_50_ in murine macrophages J774.A1; (**C**): IC_50_ in *A. castellanii* Neff cysts.

The IC_50_ and CC_50_ variations using different proportions of compounds are also shown using mixture contour plots (Figure 3). The maximum percentage for each factor (each compound) is represented in a corner of an equilateral triangle. The edges of the triangle represent the two-component blends. Each point within the triangle represents the three-component blends. The center of the triangle represents the mixture in equal proportion. Mixtures of Systane^®^ Ultra and Naviblef^®^ Daily Care presented lower IC_50_ values against trophozoites than both products separately, but this effect is even higher against cysts.

## 3. Discussion

Systane^®^ Ultra combines polyethylene glycol 400 (0.4%) with propylene glycol (0.3%) lubricants in an aqueous formulation with an HP-Guar demulcent designed to afford a prolonged lubrication to the ocular surface [15]. Both of these compounds have been reported to be effective against different bacteria species [18,19] and, on the other hand, propylene glycol also presents a remarkable preservative effect [20,21]. A previous study from our laboratory has shown the anti-*Acanthamoeba* activity of Systane^®^ Ultra and its induction of programmed cell death (PCD) mechanism [16]. In addition, the presence of a POLYQUAD^®^, which has been demonstrated as capable to activate and elevate the secretion of inflammation markers, is a disadvantage in the ocular use of Systane^®^ Ultra. However, Naviblef^®^ Daily Care does not present any described toxic preservatives on its composition but its individual IC_50_ is significantly lower than the one of Systane^®^ Ultra in the three tested strains (Table 3). Different studies have reported the activity of some Naviblef^®^ Daily Care components, such as TTO and chamomile derivatives, against *Acanthamoeba* [22,23] or AK disorders [24].

The *A. griffini* strain (genotype T3) was isolated in October 2013 from a severe case of keratitis from both a contact lens and a corneal scrape (Association to prevent Blindness in Mexico, Luis Sanchez Bulnes Hospital, Mexico City, Mexico) [25]. Chlorhexidine is the recommended treatment regimen for AK and in the study of González-Robles and colleagues this strain presented an important resistance to chlorhexidine, while it was more sensitive to voriconazole. In the present study we have reported an IC_50_ value of 7.99 ± 1.25% and 1.13 ± 0.16% of Systane^®^ Ultra and Naviblef^®^ Daily Care respectively for the same strain (Table 3).

Regarding the mixtures, the #4 mixture was the most active against trophozoites of the three *Acanthamoeba* tested strains (Figure 1). However, this #4 mixture was one of the most toxic for macrophages (Figure 2). Concerning cytotoxicity, the mixture who has presented the lowest CC_50_ value was #6, which has also presented moderate *in vitro* activity against *Acanthamoeba* spp. trophozoites and *A. castellanii* cysts. Regarding to experiment #4, 4.97% of Systane^®^ Ultra was needed to inhibit the growth of 50% of *A. castellanii* Neff trophozoites when it is used individually. However, only a 2.53% of Systane^®^ Ultra was required to produce the same effect when it was combined with Naviblef^®^ Daily Care (Table 2). Therefore, in the experiment #4, the Systane^®^ Ultra amount has been reduced to the half. In the same experiment, the percentage of Naviblef^®^ Daily Care in IC_50_ against *A. castellanii* Neff trophozoites has been also reduce three times, from 0.68% alone to 0.21% in the mixture. On the other hand, in the less cytotoxic #6 mixture, the value of Naviblef^®^ Daily Care has been also reduced, from 0.68% to 0.04% (Table 2). Despite the #6 mixture was not the most active one, both IC_50_ values against trophozoites and cysts are moderate (Table 2 and Figure 2A, respectively). Thus, it is important to highlight that the combination of Systane^®^ Ultra and Naviblef^®^ Daily Care reduce the amount of product needed to produce the same effect as their individual use. In contrast, both #2 and #11 mixtures have presented the highest cytotoxicity against the murine macrophages.

## 4. Materials and Methods

### 4.1. Chemicals

In this study the Systane^®^ Ultra ophthalmic solution and the Naviblef^®^ Daily Care cleaning foam were selected to evaluate their anti-*Acanthamoeba* *in vitro* activity (Table 3).

### 4.2. Cell Cultures

#### 4.2.1. *Acanthamoeba* Strains

In the present study three *Acanthamoeba* strains were used: (1) the type strain *Acanthamoeba castellanii* Neff genotype T4 (ATCC^®^ 30010™) and two clinical isolates: (2) *Acanthamoeba polyphaga* (Puschkarew,1913) (ATCC^®^ 30461™) genotype T4; and (3) *Acanthamoeba griffini*, genotype T3, obtained from a previous study [25]. All of them were axenically grown in PYG medium (0.75% (*w*/*v*) proteose peptone, 0.75% (*w*/*v*) yeast extract and 1.5% (*w*/*v*) glucose) containing 20 μg/mL of gentamicin (Merck KGaA, Darmstadt, Germany).

#### 4.2.2. Macrophage Cell Line

In order to evaluate the cytotoxicity in mammal cells, in this work the macrophages J774A.1 (ATCC # TIB-67) strain was used.

### 4.3. Anti-Acanthamoeba Assays

#### 4.3.1. In Vitro Effect of Systane^®^ Ultra and Naviblef^®^ Daily Care Against the Trophozoite Stage of *Acanthamoeba* Strains

The *in vitro* effect of Systane^®^ Ultra and Naviblef^®^ Daily Care against the three tested *Acanthamoeba* strains (*A. castellanii* Neff, *A. polyphaga* (ATCC^®^ 30461™) and *A. griffini*), was evaluated using a colorimetric assay based on the alamarBlue^TM^ Cell Viability Reagent (Invitrogen by Thermo Fisher Scientific), as it has previously described [7,16,26,27]. As first step, the *Acanthamoeba* culture was seeded in triplicate on a 96-well microtiter plates (50μL from a stock solution of 5 × 10^4^ cells/mL). After the trophozoites were attached to the bottom of the well, 50 μL of serial dilutions of the products were added to the 96-wells plate. As a negative control we have used *Acanthamoeba* spp. trophozoites in PYG medium. Finally, the alamarBlue^TM^ was placed onto each well at 10% as a final concentration. Then, plates were incubated during 96 h at 28 °C with a soft agitation and finally analyzed in the EnSpire^®^ Multimode Plate Reader (Perkin Elmer, Madrid, Spain) using emitted florescence at 570/585 nm. The inhibitory concentration 50 (IC_50_) was calculated by no linear regression analysis with 95% confidence limits using Sigma Plot 12.0 statistical analysis software (Systat Software). All the experiments were carried out three times each in triplicate, calculating the mean values.

#### 4.3.2. In Vitro Effect of Systane^®^ Ultra and Naviblef^®^ Daily Care Against the Cyst Stage of *A*. *castellanii* Neff Strain

*A. castellanii* Neff cysts were prepared as it has been described before [8,28]. Mature cysts were harvested and washed using PYG medium, obtaining a 5 × 10^4^ cysts/mL solution. This cysts suspension was seeded in triplicate jointly with serial dilutions of each ophthalmic solution and the plates were incubated for 168 h at 28 °C. Then, the plate was centrifuge and the supernatant, which contained each ophthalmic solution dilution, was removed and PYG was added. Finally, alamarBlue^TM^ reagent was added at 10% as a final concentration, and the plates were incubated and analyzed as it was described above.

After two weeks, an inverted microscope EVOS^®^ FL Cell Imaging System (ThermoFisher) was used to evaluate the excystment capacity after stopping the treatment. As a negative control we have used *A. castellanii* cysts incubated with PYG medium.

#### 4.3.3. In Vitro Effect Against *Acanthamoeba* spp. Trophozoites of Systane^®^ Ultra and Naviblef^®^ Daily Care Mixtures

Once the IC_50_ of each compound was calculated, the Design-Expert^®^ Software Version 10. Design-Expert^®^ software was used to provide experimental design, model building, calculation of equations, data analysis, and ideal process settings for top performance and discover optimal product formulations. In the present study, the software designed thirteen different mixtures of both compounds (Table 4), considering the IC_50_ already obtained for each one. These mixtures were carried out in double distilled sterilized water.

The *in vitro* activity of the obtained aqueous mixtures was carried out in the three *Acanthamoeba* strains as described in Section 4.3.1.

### 4.4. In Vitro Effect of Systane^®^ Ultra and Naviblef^®^ Daily Care Mixtures Against Acanthamoeba castellanii Neff Cysts Evaluated by the Alamarblue™ Colorimetric Assay

*A. castellanii* Neff cysts were prepared as it has been described before [8,27] and the protocol designed by Sifaoui and colleagues in 2018 [16] was followed. Briefly, mature cysts were harvested and washed using PYG medium, obtaining a 5 × 10^4^ cysts/mL solution. Of this cyst’s solution, 50 μL were seeded in triplicate into a 96-well microtiter plate wells, which contain 50 μL from serial dilutions of each combination. As a negative control we have used *A. castellanii* cysts incubated with PYG medium. Finally, alamarBlue^TM^ Reagent was placed into each well at 10% and the plates were incubated for 144 h at 28 °C. The plates were measured and analyzed as it has been described in previous sections.

#### In Vitro Cytotoxic Effect in Murine Macrophages J774A.1 (ATCC # TIB-67) of Systane^®^ Ultra and Naviblef^®^ Daily Care Mixtures

In order to evaluate the cytotoxicity of each new mixture of the commercialized compounds, the colorimetric assay based on the alamarBlue™ Reagent (Life Technologies, Madrid, Spain) was carried out. Fifty microliters from a stock solution of 10^5^ macrophages/mL were added in 96-well microtiter plate wells, allowing them to adhere to the bottom. Thus, 50 μL of serial dilutions of the mixtures were added to the wells. At last, the alamarBlue Reagent™ was placed into each well at 10% and the plates were incubated for 24 h at 37 °C in presence of CO_2_ at 5%. The plates were measured using the EnSpire^®^ Multimode Plate Reader as in the anti-*Acanthamoeba* assays. After all, the cytotoxic concentration 50% (CC_50_) was obtained by no linear regression analysis with 95% confidence limits using Sigma Plot 12.0 statistical analysis software as it was explained above. All the experiments were carried out three times each in duplicate, calculating the mean values.

## 5. Conclusions

In conclusion, in a mixture of Systane^®^ Ultra and Naviblef^®^ Daily Care, the maximum concentration of Systane^®^ Ultra showed an increased value of the IC_50_ against *A. castellanii* Neff trophozoites (Figure 3A), and this effect is higher against *A. castellanii* Neff cysts (Figure 3B). However, the diagram of the CC_50_ shows a clear cytotoxicity effect of the Naviblef^®^ Daily Care (Figure 3C). Naviblef^®^ Daily Care is a non-greasy and non-irritant formulation and it is recommended to be used as maintenance care, for patients that have followed the blepharitis treatment with Naviblef^®^ Intensive Care. Naviblef^®^ Daily Care continues providing protection against re-infestation from *Demodex* and maintains eyelid cleanliness. However, in the Naviblef^®^ Daily Care directions for use, they recommend rinsing the eyelashes and eyelids with warm water after each application. Tap water has been demonstrated as an important source of free-living amoeba (FLA), such as *Acanthamoeba* spp. [1,5,28]. Among the main symptoms of blepharitis, watery eyes, greasy eyelids and the appearance of skin flakes favor the establishment of pathogenic microorganisms as FLA. Both conditions could be an important risk factor to suffer AK. Despite the high toxicity effect of the present drug towards both *Acanthamoeba* and macrophages, due to its external application, it is recommended to use it as preventive tool, to eliminate possible eyelids microorganisms.

## Figures and Tables

**Table 1:** Systane^®^ Ultra and Naviblef^®^ daily care activity against *Acanthamoeba* spp.

IC_50_ (%)	Trophozoites			Cysts
	*A. Castellanii* Neff	*A. Polyphaga*	*A. Griffini*	*A. Castellanii* Neff
Systane^®^ Ultra	4.97 ± 0.71	5.98 ± 0.60	7.99 ± 1.25	34.6 ± 1.38
Naviblef^®^ Daily Care	0.68 ± 0.16	0.98 ± 0.28	1.13 ± 0.16	2.1 ± 0.04

**Table 2:** Proportion of Systane^®^ Ultra and Naviblef^®^ daily care present in each mixture IC_50_ value.

Mixture	IC_50_ (*%*) *A. Castellanii* Neff Trophozoites	Systane^®^ Ultra Proportion (%)	Naviblef^®^ Proportion (%)
#1	59.18 ± 7.56	0.59	0.06
#2	42.67 ± 3.36	0.43	0.43
#3	29.39 ± 1.57	3.53	0.03
#4	21.10 ± 1.61	2.53	0.21
#5	49.07 ± 2.56	0.49	0.27
#6	37.96 ± 3.16	2.47	0.04
#7	31.46 ± 3.07	2.05	0.31
#8	26.12 ± 1.17	3.13	0.14
#9	32.85 ± 1.91	2.13	0.18
#10	43.23 ± 2.45	1.62	0.14
#11	40.49 ± 5.11	1.52	0.31
#12	29.16 ± 3.05	2.70	0.10
#13	29.72 ± 4.23	2.75	0.23

**Table 3:** Detailed composition of the two evaluated commercial products in this study.

Systane^®^ Ultra	Naviblef^®^
Polyetilene Glycol 400 Propylene Glycol Hidroxipropilguar Sorbitol Aminomethyl Propanol Boric acid Potassium chloride Sodium chloride POLYQUAD^®^(polidronium chloride) 0.001% as preservative.	*Melaleuca alternifolia* (tea tree oil, TTO) *Anthemis nobilis* (chamomile oil) D-Panthenol Alantoine Taurine Purified water

**Table 4:** Thirteen different mixtures of Systane^®^ Ultra and Naviblef^®^ Daily Care designed by the Design-Expert^®^ Software Version 10.

Mixture	Systane^®^ Ultra (%)	Naviblef^®^ (%)	Sterile Water (%)
#1	0.0100	0.0010	0.9890
#2	0.0100	0.0100	0.9800
#3	0.1200	0.0010	0.8790
#4	0.1200	0.0100	0.8700
#5	0.0100	0.0055	0.9845
#6	0.0650	0.0010	0.9340
#7	0.0650	0.0100	0.9250
#8	0.1200	0.0055	0.8745
#9	0.0650	0.0055	0.9295
#10	0.0375	0.0033	0.9593
#11	0.0375	0.0078	0.9547
#12	0.0925	0.0033	0.9043
#13	0.0925	0.0078	0.8998
